# Supplementary material for: Application of alternative nonlinear models to predict growth curve in partridges
Source: PLoS One. 2025 Apr 15;20(4):e0321680. doi: 10.1371/journal.pone.0321680 (PMC11999107; doi:10.1371/journal.pone.0321680)
Supplement: S1 Table — (DOCX) [file pone.0321680.s001.docx]

**S1 Table. Data sets for modeling the growth curve in male and female partridges.**

| Age (day) |  | Body weight (g) | |
| --- | --- | --- | --- |
|  |  | Male | Female |
| 1 |  | 13.9 | 13.96 |
| 7 |  | 25.7 | 24.4 |
| 14 |  | 43.5 | 40.4 |
| 21 |  | 72 | 67.9 |
| 28 |  | 106 | 100 |
| 35 |  | 166 | 154 |
| 42 |  | 202 | 189 |
| 49 |  | 221 | 206 |
| 56 |  | 240 | 223 |
| 63 |  | 286 | 260 |
| 70 |  | 322 | 290 |
| 77 |  | 353 | 314 |
| 84 |  | 383 | 334 |
| 91 |  | 414 | 362 |
| 98 |  | 425 | 373 |
| 105 |  | 454 | 397 |
| 112 |  | 472 | 405 |
| 119 |  | 486 | 414 |
| 126 |  | 492 | 424 |
| 133 |  | 503 | 429 |
| 140 |  | 503 | 430 |
