# Supplementary material for: Application of alternative nonlinear models to predict growth curve in partridges
Source: PLoS One. 2025 Apr 15;20(4):e0321680. doi: 10.1371/journal.pone.0321680 (PMC11999107; doi:10.1371/journal.pone.0321680)
Supplement: Attachment — Submitted filename: Comments and suggestions.docx [file pone.0321680.s004.docx]

**Comments and suggestions:**

I believe that the following revisions can enhance the clarity, interpretability, and impact of the manuscript:

- Line 99-100: Please mention (cite) previous studies done on this issue (including the study conducted using the same data set), and justify clearly the limitation of those studies and the importance of your study.
- The sample size is too small, or not sufficient to estimate the growth parameters and to evaluate different growth models. This may lead to wrong inferences.
- There should be detailed information about the number of records per individual, the time frame and the average frequency of records.
- So, you should considered and evaluate Gompertz, Brody, Logistic, and Bertalanffy models in addition to the current growth functions and you can select the best one.
- Please define the number of parameters for each model in Table 1.
- In the figure, you should show the actual growth curve incomparision with the best-fitted growth curve, i.e. Show actual growth curve and best-fitted model in the same figure to clearly indicate the deviations.
- There is no discussion regarding the results in Table 2 (growth parameter estimates). You should discuss very well the growth parameter estimates by comparing with previous studies. The discussions mainly focus on the model comparison and estimation of absolute growth rate.
- Line 242: How do you classify traditional and modern growth function? What are the criteria to say this model is conventional or modern??? You have to justify the pros and cons of models. And also the criteria of classification (traditional and modern).
- Line 249: You said this growth model contributes to conservation of this species. How these growth models contribute to conservation of this species? Could you justify in your discussion part please?
- Line 267-277: Your discussion would be enlightening if you consider and evaluated these models in your study. Thus, please consider these models and check their goodness of fit.
- Conclusion: you should be also state the importance of validation of this model with large and frequently measured data set in addition to breed and environment.
