# Supplementary material for: Application of alternative nonlinear models to predict growth curve in partridges
Source: PLoS One. 2025 Apr 15;20(4):e0321680. doi: 10.1371/journal.pone.0321680 (PMC11999107; doi:10.1371/journal.pone.0321680)
Supplement: Attachment — Submitted filename: Revision 1 (clean version) from Reviewer.docx [file pone.0321680.s005.docx]

**Application of alternative nonlinear models to predict growth curve in partridges**

**Short title:** Alternative growth models for partridge

Navid Ghavi Hossein-Zadeh^*^

Department of Animal Science, Faculty of Agricultural Sciences, University of Guilan, Rasht, 41635-1314, Iran

**^*^Corresponding author:** Navid Ghavi Hossein-Zadeh. E-mail addresses: [nhosseinzadeh@guilan.ac.ir](mailto:nhosseinzadeh@guilan.ac.ir) or [navid.hosseinzadeh@gmail.com](mailto:navid.hosseinzadeh@gmail.com)

Tel: +98 13 33690282

Fax: +98 13 33690281

ORCID ID: [0000-0001-9458-5860](https://www.scopus.com/redirect.uri?url=http://www.orcid.org/0000-0001-9458-5860&authorId=36014893600&origin=AuthorProfile&orcId=0000-0001-9458-5860&category=orcidLink)

**Abstract**

This study aimed to describe the growth pattern in partridges using nonlinear models. Eight nonlinear mathematical functions (Bridges, Janoschek, Richards, Schumacher, Morgan, Lomolino, Sinusoidal, and Weibull) were used. Model performance was assessed and model behavior was examined during the process of fitting nonlinear regression curves. The overall goodness of fit of each model to various data profiles was assessed using the adjusted coefficient of determination, root mean square error, Akaike's information criterion, and Bayesian information criterion. The adjusted coefficient of determination values for each model are generally high, indicating that the models fit the data well overall. Based on goodness of fit criteria, the Morgan model was found to be the most appropriate function for fitting the growth curve of male and female partridges. Furthermore, the Lomolino model had the worst fit to the growth curves of male and female partridges. While the predictions of the final body weight from all the models were good, the Morgan function outperformed the others in this regard. Based on the first derivative of the Morgan model, the absolute growth rates for male and female partridges as a function of time revealed that these values gradually increased with increasing age until 42 and 35 days of age, respectively, and then declined. The Morgan function is a useful replacement for conventional growth functions when describing the growth curves of different partridge breeds.

**Keywords:** Absolute growth rate, Body weight prediction, Game bird, Goodness of fit, Mathematical function

**Introduction**

In an attempt to duplicate the advantages of animals raised in the wild, like increased productivity and better flavor, humans have had difficulty raising hunting animals in controlled environments. Scientists are searching for novel approaches to raise various game animals for meat as a result of this struggle. The two main game animals that are raised intensively these days are partridges and pheasants [1-2]. Partridges are game birds found in the wild and are primarily raised for meat production, hunting tourism, and environmental conservation [2-4]. When they reach a certain weight and age, partridges bred for hunting tourism or maintaining natural balance are released into hunting areas or the wild [5]. Partridges play a key role in the rural economy, particularly in regions where hunting tourism contributes significantly to local income. In these areas, farms specifically breed partridges for release into hunting reserves, contributing to both conservation efforts and economic sustainability. Partridges are ideal for commercial production due to their rapid growth, high productivity, efficient feed conversion, and exceptional meat quality. Partridges are considered a premium source of protein in the market due to their lean, flavorful, and nutritious meat, which has led to a global increase in demand for wild birds [6]. In captivity, partridges are an adaptable breed that work well in commercial production [7]. This has led to an increase in the number of businesses that raise partridges for meat. Partridges have similar breeding and feeding practices to other species of poultry [8]. These characteristics have led to an increase in partridge breeding and current research aims to improve productivity and growth characteristics to meet the growing demand for wild birds in the food and tourism sectors.
The potential of partridges for further domestication and genetic development also allows their productivity to be maximized and strengthens their importance in industrial poultry farming systems.

Growth is defined as changes in live weight and proportionate body component growth influenced by environmental factors and genotype; growth curves are defined as changes in growth that occur over time [9]. Growth curves are the mathematical representation of growth, and their main use is to summarize data collected at various ages. Despite their difficulty, there are a few parameters that can be understood in the context of biology [10-11]. Growth curves can be used to find the best age to slaughter, identify a measurable growth trait, learn more about the health of the living thing, and assess how selection has affected the growth curve's parameters [12-13]. Comprehending the partridge's growth curve parameters could aid scientists in initiating animal breeding initiatives [5].

When examining the growth curve of farm animals, there are a number of reasons to think about utilizing alternative models rather than traditional ones. The complex and non-linear growth trajectories seen in farm animals may not be sufficiently captured by conventional models, which frequently assume linear growth patterns. Different models are more capable of explaining differences in growth rates at various developmental stages. Alternative models allow for more customization and flexibility when fitting growth data, making it possible to capture distinct growth patterns unique to various farm animal species or breeds. By accounting for variables that can affect growth rates, such as genetic influences, environmental factors, and feed quality, alternative models may offer a more accurate depiction of growth trajectories. Alternative models might produce more accurate growth performance forecasts, empowering farmers to choose the best breeding selection, feed plans, and general management techniques. In general, alternative models can provide a more thorough and nuanced approach to analyzing farm animal growth curves than conventional models, which can enhance insights and decision-making in animal production systems [14]. Several asymptotic-mechanistic models have been developed using various mathematical functions to simulate livestock growth. These models incorporate functions based on the biological growth of the animal [15]. Parameterizing mathematical models of partridge growth and assessing alternative management and feeding strategies are necessary for optimizing partridge production systems. Nevertheless, only a few studies have described the growth curves of different partridge breeds, and most of these studies used traditional nonlinear models such as Brody, Gompertz, logistic, Von Bertalanfy, and Richards. Consequently, there are very few studies on partridge growth curves in the literature, particularly when using alternative models such as Schumacher, Morgan, Lomolino, Weibull, and sinusoidal. Therefore, this study aimed to evaluate the suitability of eight nonlinear models (Bridges, Janoschek, Richards, Schumacher, Morgan, Lomolino, Sinusoidal, and Weibull) to describe the growth pattern of partridges to incorporate alternative functions into the study and production of partridges.

**Materials and methods**

*Data sources*

Records of body weight of chukar partridges (*Alectoris chukar*) from hatching to 140 days of age are from the study of Iqbal et al. [8] and used in the current study (S1 Table). A total of 72 male and 108 female partridge chicks were weighed individually and had their wings banded. Every week, a digital scale with a sensitivity of 0.01 g was used to weigh them. Detailed information on the housing and management of the birds can be found in a study reported by Sariyel et al. [5]. Sariyel et al. [5] compared the goodness of fit of the Brody, Gompertz, Logistic, and von Bertalanffy growth curve models in this data set. Also, Iqbal et al. [8] fitted the Gompertz, Brody, Logistic, and von Bertalanffy growth models on the same data set using a Bayesian approach.

*Nonlinear models*

Table 1 displays the nonlinear models that were utilized to explain the growth curves. To model the relationship between body weight and age, the following models were fitted to the data: Bridges, Janoschek, Schumacher, Richards, Morgan, Lomolino, sinusoidal, and Weibull.

**Table 1. Functional forms of nonlinear models for describing the growth curve of partridge**

| Model | Equation | Reference |
| --- | --- | --- |
| Bridges | $y=W_{0}+\left(1-e^{-kt^{m}}\right)+\varepsilon$ | [16] |
| Janoschek | $y=a-\left(\left(a-W_{0}\right)e^{-kt^{m}}\right)+\varepsilon$ | [17] |
| Richards | $y=\frac{a}{\left(1-be^{-kt}\right)^{\frac{1}{m}}}+\varepsilon$ | [18] |
| Schumacher | $y=\frac{ab^{2}k}{\left(t+b\right)^{2}}\mathrm{e}^{\left(\frac{bkt}{t+b}\right)}+\varepsilon$ | [19] |
| Morgan | $y=ab^{k}k\frac{t^{k-1}}{\left(t^{c}+b^{k}\right)^{2}}+\varepsilon$ | [20] |
| Lomolino | $y=\frac{a}{1+b^{\log\left(\frac{k}{t}\right)}}+\varepsilon$ | [21] |
| Weibull | $y=a-\left(a-b\right)e^{-\left(\frac{k-1}{k}\right)\left(\frac{t}{IP}\right)^{k}}+\varepsilon$ | [22] |
| Sinusuidal | $y=y_{0}+a\times\sin\left(\frac{2\pi t}{b}+k\right)+\varepsilon$ | [23] |

y= represents body weight at age t (day); $\varepsilon$= Random error; a= represents asymptotic weight, which is interpreted as mature weight; and b= is an integration constant related to initial animal weight. The value of b is defined by the initial values for y and t; k= is the maturation rate, which is interpreted as weight change in relation to mature weight to indicate how fast the animal approaches adult weight; m= is the parameter that gives shape to the curve by indicating where the inflection point occurs; IP= is inflection point; W_0_= is initial body weight. For sinusoidal function, *a* is the amplitude, $y_{o}$ is the vertical offset and *k* is the phase shift. This sinusoidal function is periodic with period *b*. Also, for sinusoidal function, W_0_=y_0_+a×sin(k), and final weight is calculated by a+ y_0_.

*Statistical analysis*

The parameters of eight nonlinear models were estimated through independent fitting of the models to partridge body weight records using the NLIN and MODEL procedures in SAS [24]. Iteration techniques for fitting non-linear functions were based on the Gauss-Newton method. Before beginning this process, the NLIN and MODEL procedures evaluates the starting value specifications for the parameters. The initial values of the parameters had to be supplied for the iterative process to work. The final estimates were unaffected by the initial values that were chosen.

Using the adjusted coefficient of determination ($R_{adj}^{2}$), residual standard deviation or root means square error (RMSE), Durbin-Watson statistic (DW), Akaike's information criterion (AIC), and Bayesian information criterion (BIC), the quality of the predictions, or goodness of fit, was assessed for each model.

The following formula was used to calculate $R_{adj}^{2}$:

$$R_{adj}^{2}=1-\left[\frac{\left(n-1\right)}{\left(n-p\right)}\right]\left(1-R^{2}\right)$$

Where, the multiple coefficient of determination is denoted by $R^{2}$($R^{2}=1-\frac{RSS}{TSS}$), n denotes the number of observations (data points), p denotes the number of parameters, RSS stands for residual sum of squares, and TSS stands for total sum of squares. The $R^{2}$value is used to determine how much of the total variation around the mean of the trait can be attributed to the growth curve model. The $R^{2}$always falls within the range of 0 to 1, with the model considered satisfactory when $R^{2}$ is close to 1.

RMSE is a form of standard deviation that is calculated in the following way:

$$RMSE=\frac{RSS}{\sqrt{n-p-1}}$$

The residual sum of squares (RSS) represents the error in the data, with n being the number of observations and p being the number of parameters in the equation. The RMSE value is a critical factor in evaluating the appropriateness of growth curve models, with the most favorable model being the one with the lowest RMSE.

To assess the normality of the residuals of the fitted nonlinear models, the Shapiro-Wilk test was used. This test is commonly used to examine whether the residuals follow a normal distribution. A significant *P*-value (*P*<0.05) indicates deviations from normality. To check the assumption of constant variance in the residuals, the White’s test was also used. White's test is a common statistical test for heteroscedasticity that evaluates whether the variance of the residuals remains stable across different levels of the independent variable. A non-significant result of the White’s test (*P* > 0.05) would indicate that the homoscedasticity assumption is met, supporting the validity of the regression models. The Durbin-Watson (DW) statistic was utilized to investigate the residuals from the regression analysis for the existence of autocorrelation. Given the presence of autocorrelated residuals, it is possible that the function is inappropriate for the given data. The DW statistic has values between 0 and 4. Autocorrelation is absent when the value is close to two, positive when the value is close to zero, and negative when the value is close to four [25]. The following formula was used to determine DW:

$$DW=\frac{\sum_{t}^{n} \left(e_{t}-e_{t-1}\right)^{2}}{\sum_{t=1}^{n} e_{t}^{2}}$$

Where, $e_{t}$denotes the residual at time e, and $e_{t-1}$ presents residual at time t-1.

Using the following equation [26] (Burnham and Anderson, 2002), AIC was calculated:

$$AIC=n\times\ln\left(RSS\right)+2p$$

The AIC statistics are useful when comparing models with different levels of complexity because it adjusts the RSS based on the model's parameter count. A better fit is indicated by a lower AIC number value in the model comparison.

BIC integrates maximum likelihood (data fitting) and model selection by penalizing the (log) maximum likelihood with a term related to model complexity:

$$BIC=n\ln\left(\frac{RSS}{n}\right)+p\ln\left(n\right)$$

When comparing models, a lower BIC number denotes a better fit. In general, AIC and BIC are good criteria for comparing models that have different numbers of parameters.

Once the optimal function was chosen, the absolute growth rate, or AGR, was calculated using the function's first derivative with respect to time ($\frac{\partial y}{\partial t}$). AGR or average growth rate of animals per unit time is a useful tool for calculating the average growth rate of a population. In this case, this indicates the approximate daily weight gain during a growth phase [27]. In addition, the inflection point (IP) was calculated using the second derivative of the best function with respect to time [28-29]. As stated by Mischan et al. [28], IP is the value at which the growth model's second derivative is set to zero. The first and second derivatives of the best function were calculated using R software version 4.4.0.

**Results**

*Model parameters and goodness of fit statistics*

Table 2 displays estimated parameters of the nonlinear growth models for male and female partridges. In Table 3, goodness of fit statistics for the eight growth models fitted to body weight records are also presented. All models provided high $R_{adj}^{2}$ values, and although there were minimal differences in $R_{adj}^{2}$ values between the models, the Morgan model had the highest $R_{adj}^{2}$ value for male partridges, and the Bridges, Janoschek, Morgan, and Weibull models had the highest values for female partridges. However, the Lomolino model produced the smallest values of $R_{adj}^{2}$ for male and female partridges. DW values ranged from 0.93 (for Lomolino) to 1.39 (for sinusoidal) in male partridges and from 0.97 (for Lomolino) to 1.32 (for Morgan) in female partridges. Therefore, positive autocorrelation was observed for all models. For male and female partridges, with the exception of the Schumacher model, other functions had a normal residual distribution (P>0.05). The results of White's test examining the assumption of constant variance in the residuals confirmed residual homogeneity for all models in male partridges (P> 0.05), but the results showed that, with the exception of the sine model, other models provided residual homogeneity in female partridges (Table 3). For male partridges, the Morgan and Lomolino models provided the lowest and highest RMSE values, respectively. However, the Bridges, Janoschek, and Weibull models provided the lowest RMSE values for female partridges, but the Lomolino function had the highest value. Moreover, Morgan function had the lowest AIC and BIC values for male and female partridges, but the Lomolino model had the highest AIC and BIC values. Therefore, the Morgan model was determined to be the best function for fitting the growth curve of male and female partridges. After Morgan model, the Schumacher and sinusoidal functions provided the best fit of growth curve in male pertridges, respectively. But, after Morgan model, Schumacher model provided the best fit of growth curve in female partridges. Moreover, the Lomolino model had the worst fit to the growth curve of male and female partridges.

*Comparison of predicted growth curves*

According to Iqbal et al. [8], partridge body weights were found to increase with age during the study period, with males becoming noticeably heavier than females after 140 days of age. For male and female partridges, the Lomolino, Morgan, and sinusoidal functions provided underestimated initial body weights. However, other models predicted initial body weights close to the actual value. There existed magnitude differences between the various functions for the final or asymptotic body weights. All models provided a good prediction of final body weight, but the Morgan function provided the best prediction of final body weight compared to other models. In general, initial and final body weights predicted by different models were typically greater for male partridges compared with female partridges. Fig 1 shows predicted body weights for male and female partridges as a function of age, calculated using different nonlinear models.

*First and second derivatives of the growth curve*

Fig 2 displays the AGR values for male and female partridges as a function of time, based on the first derivative of the Morgan model. For male and female partridges, AGR values progressively rose with increasing age until 42 and 35 days of age, and then declined, respectively. For male and female partridges, based on the second derivative of the Morgan function, the IP was determined to be 42 and 35 days old, respectively.

**Table 2.** **Parameter estimates for the different growth models in partridge^*^**

| Item | Parameter^**^ |  | Model | | | | | | | |
| --- | --- | --- | --- | --- | --- | --- | --- | --- | --- | --- |
|  |  |  | Bridges | Janoschek | Richards | Schumacher | Morgan | Lomolino | Weibull | Sinusuidal |
| Male | y_0_ |  | - | - | - | - | - | - | - | 212.69 |
|  | W_0_ |  | 12.69 | 12.69 | - | - | - | - | - | - |
|  | a |  | 539.49 | 552.19 | 576.81 | 86.03 | 171817.4 | 725.79 | 552.22 | 289.62 |
|  | b |  | - | - | 0.6545 | 53.8997 | 232.5617 | 42.6009 | 12.6847 | 365.27 |
|  | k |  | 0.001021 | 0.001021 | 0.021987 | 0.158805 | 2.260343 | 79.41522 | 1.58708 | 5.4617 |
|  | IP |  | - | - | - | - | - | - | 40.94699 | - |
|  | m |  | 1.587243 | 1.587238 | -0.26496 | - | - | - | - | - |
|  | y_0_ |  | - | - | - | - | - | - | - | 154.33 |
|  | W_0_ |  | 10.29 | 10.29 | - | - | - | - | - | - |
| Female | a |  | 452.90 | 463.19 | 481.65 | 81.70 | 146162.2 | 586.25 | 463.20 | 273.82 |
|  | b |  | - | - | 0.7414 | 51.8563 | 230.3876 | 42.4601 | 10.2862 | 394.6870 |
|  | k |  | 0.001439 | 0.001439 | 0.022655 | 0.161405 | 2.20242 | 70.29801 | 1.53370 | 5.6778 |
|  | IP |  | - | - | - | - | - | - | 35.80403 | - |
|  | m |  | 1.533751 | 1.533749 | -0.33003 | - | - | - | - | - |

* Body weight records were obtained from Iqbal et al. (2019).

^**^ Model parameters are defined in Table 1.

**Table 3.** **Comparing goodness of fit for different growth curves in partridge^*^**

| Item | Statistics |  | Model | | | | | | | |
| --- | --- | --- | --- | --- | --- | --- | --- | --- | --- | --- |
|  |  |  | Bridges | Janoschek | Richards | Schumacher | Morgan | Lomolino | Weibull | Sinusuidal |
| Male | $R_{adj}^{2}$ |  | 0.9973 | 0.9973 | 0.9971 | 0.9973 | 0.9975 | 0.9962 | 0.9973 | 0.9973 |
|  | DW |  | 1.28 | 1.28 | 1.21 | 1.23 | 1.36 | 0.93 | 1.28 | 1.39 |
|  | Shapiro-Wilk (*P*-value) |  | 0.270 | 0.270 | 0.418 | 0.036 | 0.476 | 0.228 | 0.271 | 0.077 |
|  | White’s test (*P*-value) |  | 0.196 | 0.197 | 0.302 | 0.110 | 0.191 | 0.541 | 0.196 | 0.058 |
|  | RMSE |  | 8.96 | 8.96 | 9.34 | 8.92 | 8.55 | 10.70 | 8.96 | 8.89 |
|  | AIC |  | 159.58 | 159.58 | 161.33 | 158.60 | 156.84 | 166.27 | 159.58 | 159.26 |
|  | BIC |  | 99.83 | 99.83 | 101.57 | 97.80 | 96.04 | 105.46 | 99.83 | 99.50 |
| Female | $R_{adj}^{2}$ |  | 0.9973 | 0.9973 | 0.9972 | 0.9972 | 0.9973 | 0.9964 | 0.9973 | 0.9967 |
|  | DW |  | 1.31 | 1.31 | 1.25 | 1.18 | 1.32 | 0.97 | 1.31 | 1.25 |
|  | Shapiro-Wilk (*P*-value) |  | 0.261 | 0.261 | 0.529 | 0.013 | 0.244 | 0.462 | 0.262 | 0.344 |
|  | White’s test (*P*-value) |  | 0.136 | 0.136 | 0.220 | 0.085 | 0.099 | 0.374 | 0.136 | 0.041 |
|  | RMSE |  | 7.55 | 7.55 | 7.78 | 7.75 | 7.64 | 8.79 | 7.55 | 8.42 |
|  | AIC |  | 152.41 | 152.41 | 153.67 | 152.71 | 152.09 | 157.99 | 152.41 | 156.99 |
|  | BIC |  | 92.65 | 92.65 | 93.91 | 91.91 | 91.28 | 97.19 | 92.65 | 97.24 |

* Body weight records were obtained from Iqbal et al. (2019)

$R_{adj}^{2}$: Adjusted coefficient of determination; RMSE: Root means square error; DW: Durbin–Watson; AIC: Akaike information criteria; BIC: Bayesian Information Criteria

**Discussion**

This study presents a novel approach to modeling the growth curve of partridges by applying a wider range of nonlinear models than previously investigated, including less common models such as the Morgan, Lomolino, and sinusoidal functions. While traditional models such as Gompertz and logistic are widely used in poultry species, this study evaluates eight models using a comprehensive set of fitting criteria to provide a robust comparison. The results showed that the Morgan model provided the most accurate fit for both male and female partridges, an important contribution that advances growth modeling for this species. By identifying this model as the best alternative to traditional approaches, the study fills a gap in the existing literature and provides a more precise tool for predicting growth dynamics. Additionally, this research extends the application of growth models beyond commercial production to conservation efforts and highlights the dual potential for optimizing breeding programs and managing game bird populations. Thus, the study makes a significant contribution by improving the accuracy of growth predictions in partridges and expanding the scope of growth modeling in both agricultural and ecological contexts.

Accurately fitting growth curves and analyzing their parameters are essential for improving breeding and production in livestock and poultry [30]. This study aimed to identify the best model to describe partridge live weight data, which is critical for understanding growth dynamics, optimizing feeding strategies, and determining the ideal age for slaughter. Additionally, the growth curve can help evaluate genetic quality, nutrition, and management practices. The Morgan model emerged as the most appropriate function, based on its superior fit and ability to capture the growth trajectory of both male and female partridges. While all models performed well, the Morgan model’s parameters best represented the biological processes of growth, though the choice of model should always consider both statistical fit and biological relevance [31-32].

Thus far, nonlinear models have been used by some researchers to examine the growth curves of various partridge breeds. Cetin et al. [33] used the growth profiles of male and female partridges to compare the growth models (Gompertz, Logistic, and Richards). Their results showed that the Gompertz model more accurately covers the data for both male and female partridges. Also, Tholon et al. [34] used the Gompertz model in partridge research. Tholon and Queiroz [35] concluded that the Gompertz model was the most effective model to describe tinamous partridge growth due to the highest coefficients of determination, easy convergence, lower mean square predicted error, and simplicity of biological parameter interpretation. Balcioglu et al. [7] compared the growth data of male and female partridges using Gompertz, logistic, and von Bertalanffy models. Aourir et al. [36] investigated Barbary partridges (*Alectoris barbara*) using the Gompertz model. Sariyel et al. [5] analyzed growth data of partridges and concluded that the Gompertz model was most effective in determining the growth pattern. Wen et al. [30] found that the Weibull-type model explained partridge live weight data the best. Iqbal et al. [8] found that the von Bertalanffy model offered the best growth curve fit when utilizing a Bayesian approach to describe the growth of Chukar partridges. Compared with the results of Sariyel et al. [5] and Iqbal et al. [8], the results of the current study suggest a better fit of alternative nonlinear models used to describe the growth curve of partridges based on goodness of fit criteria. The Morgan model has a flexible inflection point and removes the limitation of fixed inflection point growth models such as the Gompertz equation.

The varying degrees of goodness of fit among different breeds of partridge can be attributed to differences in growth curve properties. The discrepancies in model fit seen in various studies may be the result of variations in factors such as animal breeds, body weight records, data points, and mathematical forms of the models. These variations in growth curves can be influenced by both genetic and environmental factors.

The detection of positive autocorrelations, also known as serial correlation, among the residuals in all models suggests underlying patterns not fully captured by the nonlinear growth models used in this study [25]. These autocorrelations could stem from factors such as unmodeled genetic influences, environmental variables, or time-dependent trends that affect partridge growth. Positive autocorrelation indicates that growth dynamics may be influenced by latent variables, underscoring the complexity of accurately modeling biological growth processes in this species. Importantly, the presence of autocorrelation in the residuals signals potential model misspecification or the omission of key growth-related variables, a challenge that has been similarly observed in other poultry species. This study highlights the need for advanced modeling techniques, such as the inclusion of first-order autoregressive (AR (1) models, to account for residual dependence. Implementing AR (1) corrections could improve the precision of parameter estimates and enhance the reliability of growth predictions by addressing serial dependencies in the data [37]. By directly confronting autocorrelation, the study offers a path forward for refining growth curve modeling in partridges and possibly other poultry species, emphasizing the importance of selecting models that balance fit with biological accuracy.

The analysis of AGR provides a dynamic view of how partridges grow over time, revealing periods of rapid growth as well as slower phases. Rather than relying solely on static weight measurements, AGR allows for a deeper understanding of growth trajectories, helping to identify critical growth stages such as the inflection point (IP), where growth peaks before tapering off [14, 38]. This study found that both male and female partridges reached their maximum growth rates at the IP, after which the rate of weight gain steadily declined. This trend highlights the need for targeted management interventions at key growth stages, such as adjusting feed or monitoring health, to optimize weight gain during periods of rapid growth. The later occurrence of the IP in males compared to females underscores the impact of sexual dimorphism on growth patterns in partridges. Males typically grow larger and take longer to reach full maturity, which may explain the delayed peak in their growth rate. Recognizing these differences can guide more precise nutritional strategies, allowing farmers to tailor feeding schedules to the specific needs of males and females, ultimately improving feed efficiency and reducing waste [39]. By tracking changes in AGR, producers can better understand the growth potential of their flock and make informed decisions to maximize productivity, particularly during critical growth periods when management adjustments can have the greatest impact.

**Conclusions**

In this study, the performance of eight nonlinear models in predicting the growth curves of partridges was successfully evaluated, with the aim of identifying the most suitable model for this species. Among the models analyzed, the Morgan model was found to be the most suitable for describing the growth patterns of both male and female partridges due to its superior goodness of fit criteria (e.g., RMSE, AIC, BIC, and $R_{adj}^{2}$). The Morgan model's accuracy in predicting growth curves provides valuable insights into optimizing partridge management and breeding, enabling more precise estimates of growth rates and final body weight. These results highlight the importance of selecting a robust and flexible model that can be adapted to different growth profiles in commercial and conservation environments. Future research should examine the application of this model across different breeds and environmental conditions to further validate its versatility and usefulness in poultry growth studies.

**Funding**

None.

**Ethical approval**

Not applicable.

**Conflicts of interest/Competing interests**

The author declares that he has no known competing financial interests or personal relationships that could have appeared to influence the work reported in this paper.

**Availability of data and material**

All data associated with this research are within the main text of the manuscript.

**Consent to participate**

Not applicable.

**Consent for publication**

Not applicable.

**Authors’ contributions**

**Navid Ghavi Hossein-Zadeh:** Writing – review & editing, Writing – original draft, Validation, Supervision, Project administration, Methodology, Investigation, Formal analysis, Data curation, Conceptualization.

**References**

1. Guerrini M, Panayides P, Hadjigerou P, Taglioli L, Dini F, Barbanera F. Lack of genetic structure of Cypriot Alectoris chukar (Aves, Galliformes) populations as inferred from mtDNA sequencing data. Anim Biodivers Conserv. 2007; 30:105-114.

2. Caglayan T, Kirikci K, Aygun A. Comparison of hatchability and some egg quality characteristics in spotted and unspotted partridge (*Alectoris chukar*) eggs. J Appl Poult Res. 2014; 23:244-251.

3. Cufadar Y, Olgun O, Bahtiyarca Y, Yildiz AO. Effects of dietary energy content on the performance and laying traits of the breeder chukar partridge (*Alectoris chukar*) housed in battery cages or litter floor pens. Rev Med Vet. 2010; 161:99-103.

4. Panayides P, Guerrini M, Barbanera F. Conservation genetics and management of the Chukar partridge *Alectoris chukar* in Cyprus and the Middle East. Sandgrouse. 2011; 33:34–43.

5. Sariyel V, Aygun A, Keskin I. Comparison of growth curve models in partridge. Poult Sci. 2017; 96(6):1635-1640.

6. Queiroz SA, Tholon P, Freitas EC, Bruneli FAT. Comparison of growth curve models of partridges (*Rhynchotus rufescens*) raised in captivity. Proceedings of XXII World’s Poultry Congress, pp. 8-13. Istanbul-Turkey; 2004.

7. Balcioglu MS, Kizilkaya K, Karabag K, Alkan S, Yolcu HI, Sahin E. Comparison of growth characteristics of chukar partridges (*Alectoris chukar*) raised in captivity. J Appl Anim Res. 2009; 35:21-24.

8. Iqbal F, Eyduran E, Mikail N, Sarıyel V, Huma ZE, Aygün A, Keskin İ. A Bayesian approach for describing the growth of Chukar partridges. Europ Poult Sci. 2019; 83:1-10.

9. Camdeviren H, Tasdelen B. Mono and multiphasic analysis of growth curves selected of a Japanese quail line at 5-Week’s Body Weight. Turk J Vet Anim Sci. 2002; 26:421-427.

10. Kahm M, Hasenbrink G, Lichtenberg-Frate H, Ludwig J, Kschischo M. grofit: Fitting Biological Growth Curves with R. J Stat Softw. 2010; 33:1-21.

11. Narinç D, Üçkardeş F, Aslan E. Egg production curve analyses in poultry science. World's Poult Sci J. 2014; 70:817–828.

12. Akbas Y, Taskin T, Demiroren E. Comparison of several models to fit the growth curves of Kivircik and Daglic male lambs. Turk J Vet Anim Sci 1999; 23:537-544.

13. Narinç D, Aksoy T, Karaman E, Fırat MZ. Genetic parameter estimates of growth curve and reproduction traits in Japanese quail. Poult Sci. 2014; 93:24–30.

14. Ghavi Hossein-Zadeh N. Modeling the growth curve in ducks: A sinusoidal model as an alternative to classical nonlinear models. Poult Sci. 2024; 103:103918.

15. Aygün A, Narinç D. Flexible and fixed mathematical models describing growth patterns of chukar partridges. AIP Conf Proc. 2016; 1726:020014.

16. Bridges T, Turner L, Stahly T, Usry J, Loewer O. Modeling the physiological growth of swine part I: Model logic and growth concepts. Trans ASAE. 1992; 35:1019–1028.

17. Panik MJ. Growth curve modeling: Theory and applications. John Wiley & Sons: Hoboken, NJ, USA; 2013.

18. Richards FJ. A flexible growth function for empirical use. J Exp Bot. 10(1959):290-300; 1959.

19. Schumacher FX. A new growth curve and its applications to timber yield studies. J For. 1939; 37:819-820.

20. Lopez S, France J, Dhanoa MS, Mould F, Dijkstra J. A generalized Michaelis-Menten equation for the analysis of growth. J Anim Sci. 2000; 78:1816–1828.

21. Lomolino MV. Ecology’s most general, yet protean pattern: The species–area relationship. J Biogeogr. 2000; 27:17–26.

22. Murthy DP, Xie M, Jiang R. Weibull Models; John Wiley & Sons: Hoboken, NJ, USA; 2004.

23. Arango JA, Van Vleck LD. Size of beef cows: early ideas, new developments. Genet Mol Res. 2002; 1:51–63.

24. SAS. SAS User’s Guide v. 9.1: Statistics. SAS Institute, Inc., Cary, NC; 2002.

25. Ghavi Hossein-Zadeh N. Comparison of non-linear models to describe the lactation curves of milk yield and composition in Iranian Holsteins. J Agr Sci. 2014; 152:309-324.

26. Burnham KP, Anderson DR. Model Selection and Multimodel Inference: A Practical Information-Theoretic Approach. Second edition. Springer-Verlag, Berlin, Germany; 2002.

27. Ghavi Hossein-Zadeh N. Modeling the growth curve of Iranian Shall sheep using non-linear growth models. Small Rumin Res. 2015; 130:60-66.

28. Mischan MM, Pinho SZ, Carvalho LR. Determination of a point sufficiently close to the asymptote in nonlinear growth functions. Sci Agric. 2011; 68:109–114.

29. Teixeira, GL, Fernandes TJ, Muniz JA, de Souza FAC, de Moura RS, dos Santos Melo RMP. Growth curves of campolina horses using nonlinear models. Livest Sci. 2021; 251:104631.

30. Wen Y, Liu K, Liu H, Cao H, Mao H, Dong X, Yin Z. Comparison of nine growth curve models to describe growth of partridges (*Alectoris chukar*). J Appl Anim Res. 2019; 47(1):195-200.

31. Darmani Kuhi H, Ghavi Hossein-Zadeh N, López S, Falahi S, France J. Sinusoidal function to describe the growth curve of dairy heifers. Anim Prod Sci. 2019; 59:1039-1047.

32. Dogan N, Emre K, Mehmet ZF, Tulin A. Comparison of non-linear growth functions to describe the growth in Japanese quail. J Anim Vet Adv. 2010; 9:1961-1966.

33. Cetin M, Sengul T, Sogut B, Yurtseven S. Comparison of growth models of male and female partridges. J Biol Sci. 2007; 7:964-968.

34. Tholon P, Freitos EC, Stein MS, Queiroz SA. Genetic parameters estimates to Gompertz growth parameters fitted to partridges (*Rhynchotus rufensces*) raised in captivity. Proceedings of 12^th^ International Europan Poultry Conferences, pp. 1–4 and 10– 14. Verona, Italy; 2006.

35. Tholon P, Queiroz SA. Models for the analysis of growth curves for rearing tinamous (*Rhynchotus rufensces*) in captivity. Braz J Poult Sci. 2007; 1:23-31.

36. Aourir M, El Abbasi A, Znari M. patterns in Barbary partridges (*Alectoris barbara*) originated from low-and high elevations in West central Morocco. Avocelta. 2014; 38:45-51.

37. Silva WS, Fernandes FA, Muniz FR, Muniz JA, Fernandes TJ. Eucalyptus Grandis X Eucalyptus Urophylla growth curve in different site classifications, considering residual autocorrelation. Braz J Biom. 2021; 39(1):122–138.

38. Boonkum W, Chankitisakul V, Kananit S, Kenchaiwong W. Heat stress effects on the genetics of growth traits in Thai native chickens (Pradu Hang dum). Anim Biosci. 2024; 37(1):16-27.

39. Müsse J, Louton H, Spindler B, Stracke J. Sexual dimorphism in bone quality and performance of conventional broilers at different growth phases. Agriculture. 2022; 12:1109.

**Fig 1.** **Predicted body weights as a function of age, determined using different nonlinear models for male and female partridges.**

**Fig 2. Absolute growth rate (AGR) as a function of age for male and female partridges based on the Morgan model.**
