# Supplementary material for: Application of alternative nonlinear models to predict growth curve in partridges
Source: PLoS One. 2025 Apr 15;20(4):e0321680. doi: 10.1371/journal.pone.0321680 (PMC11999107; doi:10.1371/journal.pone.0321680)
Supplement: Attachment — Submitted filename: Response to Reviewers.docx [file pone.0321680.s008.docx]

With sincere thanks to the respective Academic Editor and reviewers for their constructive comments, I am pointing to the changes I made to the revised manuscript.

**************************************************

**Journal Requirements:**

Please review your reference list to ensure that it is complete and correct. If you have cited papers that have been retracted, please include the rationale for doing so in the manuscript text, or remove these references and replace them with relevant current references. Any changes to the reference list should be mentioned in the rebuttal letter that accompanies your revised manuscript. If you need to cite a retracted article, indicate the article’s retracted status in the References list and also include a citation and full reference for the retraction notice.

**AU:** All references were checked for completeness and correctness as recommended. There are no retracted papers in the citations and reference list of this manuscript.

***********************************************************

**Reviewer 6:**

- I suggest interpreting the Durbin-Watson test by the p-value, as you did in the other tests of the residual analysis (Shapiro-Wilk and White's). As it is presented, how will the reader know if the test was significant? In other words, how close to 2 should the DW value be to say that Autocorrelation is absent? This makes it easier for the reader to understand. In the R software, simply use the durbinWatsonTest() function.

**AU:** Thank you for your excellent suggestion. The information on the use of P-values to interpret the Durbin-Watson test results was added to the text as recommended (Lines 189-192).

- Table 3: As I mentioned, I suggest also including the p-value for the Durbin-Watson test, as you did for the Shapiro-Wilk and White's tests. Or, at least indicate the critical heat of the test, above which value would be Autocorrelation absent (I imagine it to be something like 1.5 < DW < 2.5 depending on the significance level adopted in the test).

**AU:** The P-values of the Durbin-Watson test were added to Table 3 as recommended.

- Figures: Improve the resolution of the figures significantly, especially Figure 1.

**AU:** Both figures are currently being produced by PACE, provided by PLOS ONE, to prepare high quality figures for publication.
